# Takotsubo Syndrome: Uncovering Myths and Misconceptions

**DOI:** 10.1007/s11883-021-00946-z

**Published:** 2021-07-16

**Authors:** Victoria L. Cammann, Michael Würdinger, Jelena R. Ghadri, Christian Templin

**Affiliations:** Andreas Grüntzig Heart Catheterization Laboratories, Department of Cardiology, University Heart Center, University Hospital Zurich, Raemistrasse 100, 8091 Zurich, Switzerland

**Keywords:** Takotsubo syndrome, Broken heart syndrome, International Takotsubo Registry, Risk stratification, Outcome

## Abstract

**Purpose of Review:**

Takotsubo syndrome (TTS) was described in Japan 3 decades ago to affect predominately postmenopausal women after emotional stress. This history is the basis of commonly held beliefs which may contribute to the underdiagnosis and misperception of TTS.

**Recent Findings:**

TTS affects not only women, but can be present in both sexes, and can appear in children as well as in the elderly. TTS is characterized by unique clinical characteristics with morphological variants, and incurs a substantial risk for recurrent events and adverse outcomes. Physical triggers are more common than emotional triggers and are major disease determinants. TTS seems not to be completely transient as patients report ongoing chest pain, dyspnea, or fatigue even after months of the acute event.

**Summary:**

Knowledge of the clinical features and outcomes of TTS patients has evolved substantially over the past decades. The heterogeneous appearance of TTS needs to be recognized in all medical disciplines to maximize therapy and improve outcomes.

## Introduction

**Fig. 1:**
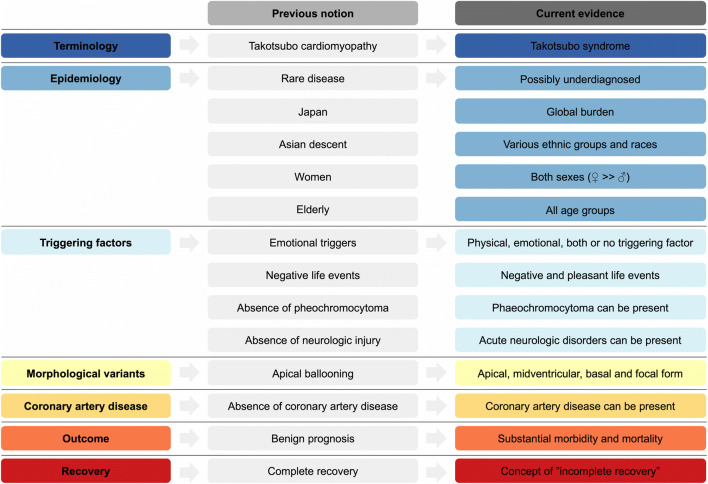
Evolution and paradigm shifts of takotsubo syndrome.

In 1991, Sato and colleagues from the Hiroshima City Hospital reported 5 cases of women who presented with an unusual left ventricular (LV) wall motion pattern after severe emotional stress [1, 2]. The unique morphological feature of the LV, with shared similarities to a Japanese octopus trap (narrow neck and wide bottom), led the Japanese cardiologists to term this cardiac entity “takotsubo.” Since the seminal observation, takotsubo syndrome (TTS) has emerged as an important differential diagnosis of patients presenting with acute chest pain. This review focuses on widely held myths and misconceptions to amplify perception of TTS (Fig. 1).

## Definition

Takotsubo syndrome is an acute heart failure syndrome characterized by LV systolic dysfunction [3–5]. Patients with TTS may present with typical acute coronary syndrome (ACS) symptoms including chest discomfort or dyspnea, ST-segment deviations on electrocardiogram, and cardiac biomarker abnormalities [6]. Cardiac catheterization is the cornerstone of diagnosis to differentiate TTS from other etiologies such as ACS or myocardial infarction without obstructive coronary arteries (MINOCA) [7–9]. The pathophysiology of TTS is not well understood, and enhanced sympathetic stimulation and elevated circulating catecholamines were first suggested to be major mediators [10]. The pathophysiology likely involves a complex interplay of central autonomic dysfunction, hormones, multi-vessel coronary spasm, microvascular dysfunction, and inflammation [11•].

## Terminology

Over the past years, more than 70 names have been introduced how to call this cardiac condition, further underpinning the diversity and heterogeneity of features [12, 13]. The most popular names are “broken heart syndrome,” “stress cardiomyopathy,” or “apical ballooning syndrome.” None of them universally reflects the spectrum of presentation and morphological features and includes inherent limitations which may have contributed to misconceptions and misdiagnosis. The term “broken heart syndrome” implies that negative emotional or psychological triggering factors such as the loss of a beloved person are mandatory to be present. Likewise, the term “stress cardiomyopathy” suggests that the disease must occur in the presence of a triggering event. “Apical ballooning” implies that the classical apical ballooning is the only existing morphological variant of TTS [12, 14••].

TTS was originally designated as a primary acquired cardiomyopathy by the American Heart Association and as an unclassified cardiomyopathy by the European Society of Cardiology [15, 16]. With more advanced understanding of the disease, it has become apparent that TTS does not share common characteristics of non-ischemic cardiomyopathies and that it might more likely represent a microvascular form of ACS [17]. Based on clinical experience and advances in clinical science and in acknowledgment of the Japanese investigators, there is now international consensus that the most suitable name is “takotsubo syndrome” [14••].

## Epidemiologic Features

An estimated 4% of patients who are admitted with signs and symptoms of an ACS are diagnosed as TTS [18–20]. The true incidence of TTS is likely to be higher due to underreporting and misdiagnosis of TTS cases [21]. TTS shows a strong predilection for females (9:1 female-to-male ratio), and more than 80% of patients involve females over the age of 50 years [6, 22, 23]. The mean age of women at TTS diagnosis is around 67 years and 63 years for men [6]. TTS was completely underrecognized in most parts of the world until first reports from the USA and France were published nearly 10 years after the initial description in Japan [24, 25]. TTS now represents a global disorder affecting both sexes, all age groups, and various ethnic groups and races.

## Life Event Triggers for TTS

TTS is characteristically triggered by emotional or physical inciting events. However, absence of triggering factors does not preclude the diagnosis of TTS. Negative emotional life events have unequivocally been acknowledged as the prototypical provocation for TTS. In earlier years, physical factors (e.g., traumata, surgery, or medical conditions) were not known to trigger TTS. Similarly, it was inconceivable that TTS could also occur in the absence of stressful life events or in the presence of both emotional and physical stress. In the InterTAK Registry, only 28% of patients had emotional triggering factors [6]. Physical triggering factors were more prevalent than emotional triggers and were more common in men. Notably, one-third of patients had the absence of identifiable triggering events [6]. In 2016, a novel conceptual entity termed as the “happy heart syndrome” was described adding pleasant life events to the heterogeneous spectrum of triggering events [26]. This concept has presented an additional paradigm shift beyond the commonly accepted spectrum of inciting events.

## Pheochromocytoma as a Trigger for TTS

Pheochromocytoma is a catecholamine-releasing adrenal tumor [27]. The incidence of pheochromocytoma is generally low (<1% per 100,000 patients’ years) but has increased due to intensified imaging and biochemical testing over recent years [28]. The clinical presentation of patients with pheochromocytoma encompasses an extremely broad spectrum and can range from hypertensive crisis to severe hypotension with shock [27, 29]. Nearly all diagnostic criteria for TTS have excluded pheochromocytoma as a trigger [30–35]. TTS due to pheochromocytoma has comparable clinical features and imaging and histopathological findings to TTS triggered by physical or emotional stress [36, 37]. Based on current evidence, there is no obvious reason to exclude excessive catecholamine release from pheochromocytoma as a possible triggering factor for TTS [37, 38••].

## Neurologic Disorders as Triggers for TTS

Cardiac abnormalities are frequently observed after neurologic disorders such as stroke, subarachnoid hemorrhage, or epilepsy [39–42]. The mechanism of developing TTS may be related to increased concentrations of catecholamines after neurologic disorders, which may cause myocardial injury [43, 44]. The initial version of the Mayo Clinic Diagnostic criteria excluded the presence of head trauma or intracranial bleeding for diagnosis of TTS [32]. However, advances in clinical science have uncovered that neurologic disorders are important triggering factors of TTS and constitute 16% of all physical triggers [6, 45–47]. Intriguingly, the prevalence of (acute or past) neurologic disorders is 2 times higher in TTS patients compared to age- and sex-matched controls with ACS [6]. More recently, functional magnetic resonance imaging studies demonstrated alterations of brain regions (central autonomic network), further highlighting the importance of the brain-heart axis in the development of TTS [48–50]. Thus, the association between neurologic disorders and TTS is not only for the cardiologists but also for all clinicians to recognize.

## Diagnosis

Diagnostic criteria for TTS were proposed from multiple centers in various countries [30, 33–35, 51, 52]. In 2018, the InterTAK Diagnostic Criteria were developed with consensus from 36 experts to provide standardized diagnostic criteria incorporating the most recent and updated evidence available for TTS [••]. The InterTAK Diagnostic Criteria added neurologic disorders, the presence of coronary artery disease, and pheochromocytoma as inclusion criteria to improve diagnosis of TTS (Table 1) [••]. TTS diagnosis is particularly challenging in “special populations” such as children, in patients with neurologic disorders, and in patients without ECG deviations. In such cases, serial measurements of cardiac biomarkers and echocardiography should be performed to increase the sensitivity of TTS. Coronary angiography with ventriculography is the cornerstone of diagnosis to exclude critical coronary lesions which are the culprits for wall motion abnormalities [8]. Coronary computed tomography can be performed in patients with high pre-test probability of TTS. Cardiac magnetic resonance imaging is especially valuable in patients with red flags of myocarditis. If the focal form is present, cardiac magnetic resonance imaging is recommended to rule out acute myocardial infarction (AMI) or myocarditis and to rule in TTS [53]. Absence of late-gadolinium enhancement and presence of myocardial edema are suggestive for TTS [54].

**Table 1:** International Takotsubo Diagnostic Criteria (InterTAK Diagnostic Criteria) for takotsubo syndrome [14••]

1. Patients show transient^a^ left ventricular dysfunction (hypokinesia, akinesia, dyskinesia) presenting as apical ballooning or midventricular, basal, or focal wall motion abnormalities. Right ventricular involvement can be present. Besides these regional wall motion patterns, transitions between all types can exist. The regional wall motion abnormality usually extends beyond a single epicardial vascular distribution; however, rare cases can exist where the regional wall motion abnormality is present in the subtended myocardial territory of a single coronary artery (focal TTS).^b^ 2. An emotional, physical, or combined trigger can precede the takotsubo syndrome event, but this is not obligatory. 3. Neurologic disorders (e.g., subarachnoid hemorrhage, stroke/transient ischemic attack, or seizures) as well as pheochromocytoma may serve as triggers for takotsubo syndrome. 4. New ECG abnormalities are present (ST-segment elevation, ST-segment depression, T-wave inversion, and QTc prolongation); however, rare cases exist without any ECG changes. 5. Levels of cardiac biomarkers (troponin and creatine kinase) are moderately elevated in most cases; significant elevation of brain natriuretic peptide is common. 6. Significant coronary artery disease is not a contradiction in takotsubo syndrome. 7. Patients have no evidence of infectious myocarditis.^b^ 8. Postmenopausal women are predominantly affected.	

^a^Wall motion abnormalities may remain for a prolonged period of time or documentation of recovery may not be possible. For example, death before evidence of recovery is captured.

^b^Cardiac magnetic resonance imaging is recommended to exclude infectious myocarditis and diagnosis confirmation of takotsubo syndrome.

Clinically, TTS is indistinguishable from AMI. The InterTAK Diagnostic Score is a validated model for differentiation of TTS and ACS with high specificity and sensitivity [18]. Low InterTAK Diagnostic Score values indicate a high pre-test probability for ACS and high InterTAK Diagnostic Score values indicate a high pre-test probability for TTS [18]. The InterTAK Diagnostic Score is available online at www.takotsubo-registry.com.

## Morphological Variants

Takotsubo syndrome is classified in 4 distinctive phenotypes, depending on the region of LV wall motion abnormalities [27]. In the most typical (apical) TTS variant, which constitutes around 80% of cases, there is apical akinesis and basal hypercontractility [6, 27]. Atypical TTS types include the midventricular, basal, and focal form, and are present in 15%, 2%, and 2% of cases respectively, [6, 27]. Over the past years, atypical TTS variants have been more frequently observed, indicating that awareness of such variants has expanded [27]. In all TTS types except focal, wall motion abnormalities are not solely confined to a single coronary artery vessel [••]. Right ventricular involvement is present in approximately one-fourth of TTS cases [55]. The presence of right ventricular involvement has been suggested to present a severity marker for a more eventful clinical course and worse outcomes [56].

## Coronary Artery Disease

Anterior myocardial infarction with wrap-around left anterior descending (LAD) artery can resemble an apical TTS phenotype. The “apical nipple sign” can help to distinguish TTS from anterior ST-segment elevation myocardial infarction (STEMI) in such cases [57].

One strongly held belief was that the diagnosis of TTS requires the absence of coronary artery disease (CAD). In the InterTAK Registry, 15.3% of patients had single-vessel and 7.8% of patients had multi-vessel disease, in whom the underlying CAD could not explain the wall motion abnormalities [58]. If TTS is suspected and CAD is present, a thorough comparison of angiography and left ventriculography should be performed to assess a potential perfusion-contraction mismatch [9]. This is of particular importance, as the limited knowledge on the coexistence of CAD and TTS might contribute to a substantial underdiagnosis of TTS. Whether CAD in TTS is causal or a bystander, and whether ad hoc percutaneous coronary intervention (PCI) or staged PCI should be performed in patients with TTS, warrants further investigation.

## Prognosis

The short- and long-term sequelae of TTS were initially underestimated, because it was assumed that TTS is a harmless and self-healing condition. Recent data have demonstrated that TTS is associated with substantial risk of morbidity and mortality, with rates comparable to AMI [6, 59]. The clinical course of TTS can be complicated by cardiac arrest, cardiogenic shock, or malignant arrhythmias [60–65]. The rate of death is 6% per patient-year and the major adverse cardiac and cerebrovascular event (MACCE) rate is 10% per patient-year [6].

Extra-cardiac predictors for adverse outcomes are male sex, physical triggering factors, and acute neurologic disorders. Cardiac predictors for adverse outcomes are troponin levels over 10 times the upper limit of the normal range, high BNP values, left ventricular ejection fraction (LVEF) below 45%, moderate-to-severe mitral regurgitation, and right ventricular involvement [6, 65, 66]. Prognosis of TTS after emotional stress factors is generally favorable. Mortality is likely attributable to a combination of coexisting medical conditions and TTS [59, 67]. Mortality is doubled for patients with physical triggers compared to patients with ACS [59]. The InterTAK Prognostic Score was established for risk prediction and incorporates covariates strongly associated with prognosis such as demographics, triggering factors, hemodynamics, and comorbidities [68].

The recurrence rate is estimated at 2% per patient-year, occurring over a period of 30 days to 10 years after documented wall motion recovery of the index event. Initially, the previously affected area was thought to be protected from recurrence, analogous to regional ischemic preconditioning [69]. However, data from multicenter registries consistently demonstrated that the same myocardial area can be affected at the index and recurrent event. Different wall motion patterns and triggering factors are observed in 20–30% of cases [70, 71]. Neurologic or psychiatric disorders are independent predictors for TTS recurrence [71, 72].

## Transience and Recovery of Wall Motion Abnormalities

Transience of wall motion abnormalities and recovery of LV function have historically been acknowledged as central concepts of TTS [3, 32]. In the vast majority of cases, recovery of wall motion abnormalities and normalization LVEF can be observed within days to months after the acute event [73]. Factors associated with late recovery are male sex, LVEF below 45%, and acute neurologic disorders [74].

However, ongoing chest discomfort, dyspnea, fatigue, and reduced exercise capability beyond the acute phase can persist for months after the TTS event despite normalization of myocardial function [75]. Structural and metabolic alterations of the myocardium were observed in the long-term, suggesting that TTS may be a persistent heart failure phenotype [76]. This novel concept of “incomplete recovery” may open an avenue for further research to uncover the mechanistic insights involved.

## Management

Contemporary management of TTS is mainly empiric and relies on a combination of heart failure treatment and therapy of pre-existing medical conditions. Randomized controlled trials have not been performed yet, and data from observational studies or case series have partially shown conflicting results [5]. In 2018, a management algorithm on acute and long-term management as well as management of complications was issued by an international expert panel (Level of evidence C) to provide management approaches for patients with TTS [38••].

QT prolongation can be found in a substantial number of cases and can predispose the risk for development of torsade de pointes tachycardia [77–79]. QT-prolonging drugs need to be strictly avoided in TTS and monitoring for at least 48 h is recommended. β-adrenergic blockade is the standard treatment of choice for tachycardiac rhythms and temporary pacing can be considered in patients with atrioventricular block. Implantable cardioverter defibrillator (ICD) therapy to prevent sudden cardiac death in patients with severely reduced EF is not recommended due to normalization of ECG changes and LVEF within weeks after the event. Wearable defibrillators may be an option for patients with severely reduced LVEF [38••, 80].

Cardiogenic shock in TTS can either develop from severe pump failure or left ventricular outflow tract obstruction (LVOTO) [5, 81]. Catecholamines for hemodynamic stabilization should be avoided or administrated with great caution given the putative involvement in the pathophysiology [82]. Catecholamine therapy can aggravate LVOTO leading to further deterioration of cardiogenic shock. The calcium sensitizer levosimendan has been suggested to present a therapeutic alternative in such cases [83]. Mechanical support with microaxial pumps (Impella) might also represent a therapeutic option for TTS patients with cardiogenic shock [84, 85]. The effectiveness of mechanical support with Impella is unknown, and all available data is based on case reports, which report excellent outcomes but might suffer from inherent publication bias [86].

LV thrombus may develop in regions of akinetic segments [87, 88]. In these patients, therapeutic anticoagulation might be considered for 3 months [88]. Unlike in patients with cardiac aneurysm, lifelong anticoagulation is not recommended since wall motion abnormalities are transient [89]. Anticoagulation may also be considered in TTS patients with severe wall motion abnormalities to prevent LV thrombus formation.

Observational studies and meta-analyses consistently reported that short- and long-term treatment with β-blockers is not beneficial for mortality reduction nor recurrence prevention [6, 72, 90–92]. The use of angiotensin-converting enzyme (ACE) inhibitors or angiotensin receptor blockers (ARB) was associated with a survival benefit and less recurrence of events [6, 72]. Cardiac rehabilitation may be beneficial for improvement of quality of life and to reduce episodes of ongoing chest pain [93]. CAD should be treated as directed by guideline [94]. Psychiatric counseling may be valuable for coexistent comorbidities and triggers.

## The Value of Registries

In the past decades, we have learned major lessons from several single center and multicenter registries, which have contributed to an advanced understanding of the condition. The International Takotsubo Registry (InterTAK Registry) was established at the University Hospital Zurich, Switzerland, in 2011 to represent a unique multicenter database to raise awareness, provide insights regarding clinical features and outcomes, develop risk stratification tools for diagnosis and prognosis, and guide management for patients with TTS [95, 96]. In 2015, the initial report of the InterTAK investigators was published in the *New England Journal of Medicine* extensively describing clinical features and outcomes of patients with TTS using data from 1750 patients derived from 26 sites in 9 countries [6]. Currently, more than 3500 patients from 56 active sites from 18 countries are included in the registry, making the InterTAK Registry the largest database for TTS patients.

## Conclusion

Three decades after the seminal description of TTS, the knowledge base on clinical features, risk factors, and outcomes has remarkably expanded, and many myths and misconceptions were uncovered. Future investigations are needed to gain a better understanding of the pathophysiological mechanisms and to provide tailored therapeutic approaches to improve outcomes and prevent recurrences.
